# Supplementary material for: Manipulating hydrogen bond dissociation rates and mechanisms in water dimer through vibrational strong coupling
Source: Nat Commun. 2023 Jun 14;14:3527. doi: 10.1038/s41467-023-39212-y (PMC10267182; doi:10.1038/s41467-023-39212-y)
Supplement: Supplementary file 1 — Supplementary Information [file 41467_2023_39212_MOESM1_ESM.pdf]

Supplementary Information for Manipulating hydrogen bond dissociation rates and mechanisms in water dimer through vibrational strong coupling

Qi Yu[1,2*] and Joel M. Bowman[2]

E-mail: qyu28@emory.edu

[1]Department of Chemistry, Yale University, New Haven, Connecticut, 06520, U.S.A.

[2]Department of Chemistry, Emory University and Cherry L. Emerson Center for Scientific Computation, Atlanta, Georgia, 30322, U.S.A.

Supplementary Note 1. N-mode representation

In N-mode representation, the effective potential can be written as:

$$
\begin{aligned}
V^{\text{eff}}(\mathbf{Q},\mathbf{q}) &= \sum_i V_i^{(1)}(Q_i) + \sum_i V_i^{(1)}(q_i) + \sum_{i,j} V_{ij}^{(2)}(Q_i,Q_j) + \sum_{i,j} V_{ij}^{(2)}(Q_i,q_j) \\
&+ \sum_{i,j} V_{ij}^{(2)}(q_i,q_j) + \sum_{i,j,k} V_{ijk}^{(3)}(Q_i,Q_j,Q_k) + \sum_{i,j,k} V_{ijk}^{(3)}(Q_i,Q_j,q_k) + \sum_{i,j,k} V_{ijk}^{(3)}(Q_i,q_j,q_k) \\
&+ \sum_{i,j,k} V_{ijk}^{(3)}(q_i,q_j,q_k) + \sum_{i,j,k,l} V_{ijkl}^{(4)}(Q_i,Q_j,Q_k,Q_l) + \sum_{i,j,k,l} V_{ijkl}^{(4)}(Q_i,Q_j,Q_k,q_l) \\
&+ \sum_{i,j,k,l} V_{ijkl}^{(4)}(Q_i,Q_j,q_k,q_l) + \sum_{i,j,k,l} V_{ijkl}^{(4)}(Q_i,q_j,q_k,q_l) + \sum_{i,j,k,l} V_{ijkl}^{(4)}(q_i,q_j,q_k,q_l) \\
&+ \cdots ,
\end{aligned}
\tag{1}
$$

where

$$V_i^{(1)} = V(Q_i; Q_{j\neq i} = 0) \tag{2}$$

$$V_{ij}^{(2)} = V(Q_i, Q_j; Q_{k\neq i,j} = 0) - V_i^{(1)} - V_i^{(1)} \tag{3}$$

$$V_{ijk}^{(3)} = V(Q_i, Q_j, Q_k; Q_{l\neq i,j,k} = 0) - V_{ij}^{(2)} - V_{ik}^{(2)} - V_{jk}^{(2)} - V_i^{(1)} - V_j^{(1)} - V_k^{(1)} \tag{4}$$

$$
\begin{aligned}
V_{ijkl}^{(4)} = V(Q_i, Q_j, Q_k, Q_l; Q_{m\neq i,j,k,l} = 0) - V_{ijk}^{(3)} - V_{ijl}^{(3)} - V_{ikl}^{(3)} - V_{jkl}^{(3)} - V_{ij}^{(2)} \\
- V_{ik}^{(2)} - V_{il}^{(2)} - V_{jk}^{(2)} - V_{jl}^{(2)} - V_{kl}^{(2)} - V_i^{(1)} - V_j^{(1)} - V_k^{(1)} - V_l^{(1)}
\end{aligned}
\tag{5}
$$

$V_i^{(1)}(Q_i)$ and $V_i^{(1)}(q_i)$ are the one-mode potentials for the molecular normal mode and the cavity mode, respectively, i.e., the 1D cut through the effective potential along each mode. $V_{ij}^{(2)}(Q_i, Q_j)$, $V_{ij}^{(2)}(q_i, q_j)$, and $V_{ij}^{(2)}(Q_i, q_j)$ are the intrinsic 2-mode potentials for pairs of molecular normal modes, pairs of cavity modes, and pairs composed of a molecular normal mode and a cavity mode. Similar definitions apply to $V^{(3)}$ and $V^{(4)}$.

Similarly, the the dipole moment of the molecule is expressed such that

$$\mu(\mathbf{Q}) = \sum_i \mu_i^{(1)}(Q_i) + \sum_{i,j} \mu_{ij}^{(2)}(Q_i, Q_j) + \sum_{i,j,k} \mu_{ijk}^{(3)}(Q_i, Q_j, Q_k) + \cdots , \tag{6}$$

The explicit expressions for the one-mode, 2-mode, and 3-mode dipole moments follow the same pattern as the potential components in Supplementary Eq. (2)-(5). Interested readers are referred to the literature.[1,2]

Supplementary Table 1: Harmonic frequencies (cm^{-1}) and double harmonic intensity ($km{\cdot}mol^{-1}$, in parentheses) of $(H_2O)_2$.

Mode	MP2/aVTZ	CCSD(T)-F12b/aVTZ	PES	Assignment
1	127 (35)	128	125 (82)	Water wag
2	147 (65)	139	134 (145)	Water torsion
3	155 (131)	153	149 (145)	H-bond torsion
4	184 (149)	186	185 (136)	O-O stretch
5	360 (51)	353	354 (53)	Out-of-plane bend
6	630 (91)	617	612 (96)	In-plane bend
7	1628 (87)	1648	1648 (90)	Bend (D)[a]
8	1650 (36)	1669	1670 (36)	Bend (A)[a]
9	3719 (297)	3751	3752 (262)	H-bonded OH stretch (D)
10	3814 (11)	3828	3830 (7)	Sym OH stretch (A)
11	3915 (115)	3915	3914 (95)	Free OH stretch (D)
12	3935 (97)	3935	3935 (77)	Asym OH stretch (A)

[a] Donor water (D), Acceptor water (A)

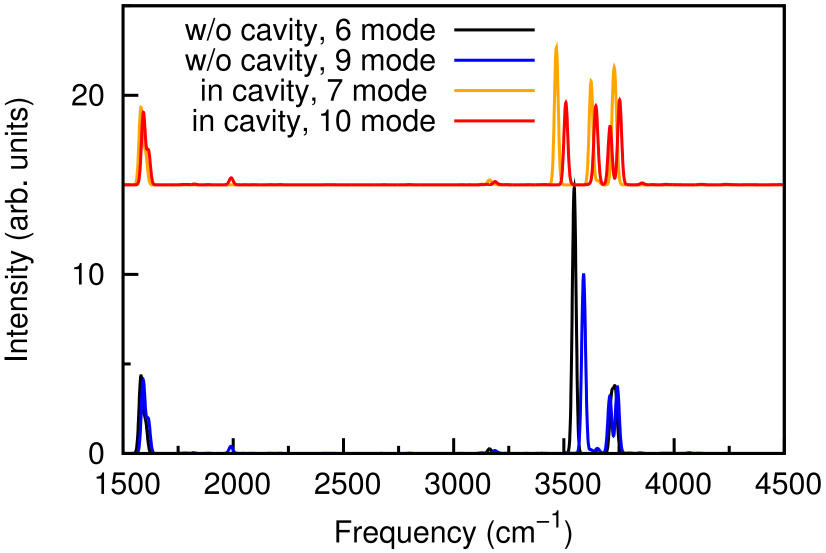

Supplementary Figure 1: Infrared spectra of $(H_2O)_2$ with and without the inclusion of three low-frequency modes (O-O stretch, out-of-plane bend, and in-plane bend), for the $(H_2O)_2$ in and out of the cavity. 6 mode calculation includes only Mode 7-12 while 9 mode calculation includes Mode 4-12 as listed in Supplementary Table 1. 7 mode and 10 mode calculations are similar to 6 mode and 9 mode calculations except for the additional inclusion of the cavity mode. A single cavity mode with a frequency of 3547 cm^{-1} is considered and the coupling strength factor g is set as 0.005.

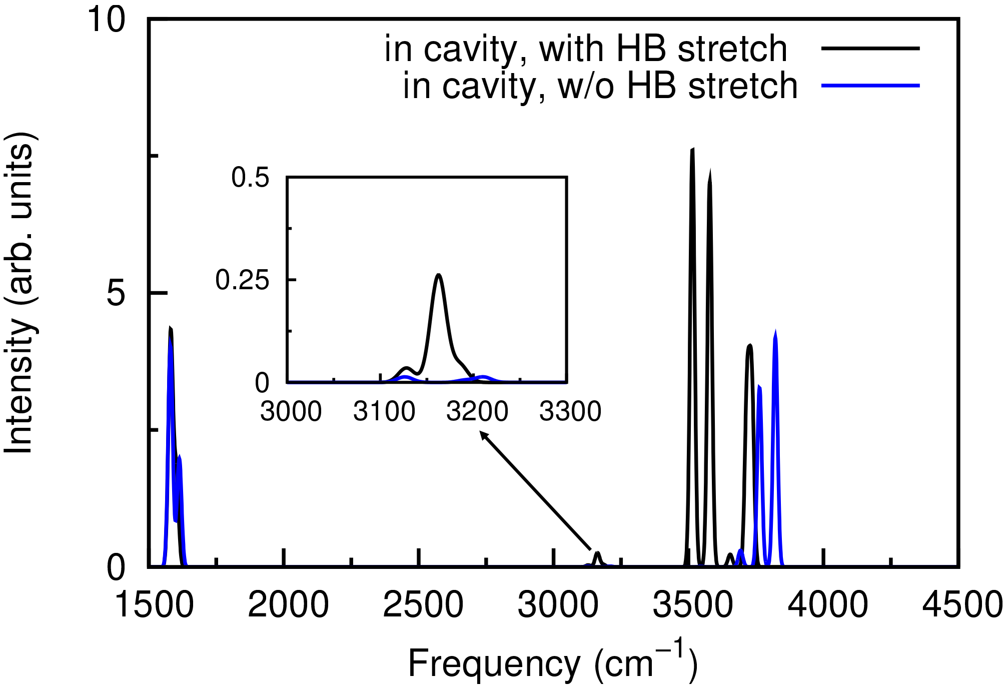

Supplementary Figure 2: Infrared spectra of $(H_2O)_2$ with and without the inclusion of HB stretch, for the $(H_2O)_2$ in the cavity. A single cavity mode with a frequency of 3547 cm^{-1} is considered and the coupling strength factor g is set as 0.002.

Supplementary Table 2: Cavity vibrational self-consistent field/configuration interaction (cav-VSCF/VCI) state energies and leading vibrational configuration interaction (VCI) coefficient(s) for calculations in Supplementary Figure 1 with and without the inclusion of three low-frequency modes (O-O stretch, out-of-plane bend, and in-plane bend). "/" indicates the VCI coefficients smaller than 0.005. "HB str", "free str", and "sym str" indicate the hydrogen bonded OH stretch, free OH stretch, and symmetric OH stretch respectively.

	w/o cavity, 6 mode					w/o cavity, 9 mode				
Energy (cm^{-1})	3162	3547	3547[a]	3655	3716	3185	3588	3592	3563	3743
VCI coeff (cavity)	/	/	1.0	/	/	/	/	/	/	/
VCI coeff (HB str)	0.15	0.97	/	0.08	0.09	0.13	0.67	0.43	/	0.02
VCI coeff (free str)	0.04	0.16	/	/	0.99	0.03	0.02	/	/	0.92
VCI coeff (sym str)	0.04	/	/	0.98	/	/	0.03	0.04	0.78	/
VCI coeff (bend overtone[b])	0.76	0.15	/	/	0.05	0.82	0.10	0.05	/	0.04
VCI coeff (low-freq+bend[c])	/	/	/	/	/	0.37	0.30	0.64	0.26	/
VCI coeff (low-freq[d])	/	/	/	/	/	/	0.32	/	0.25	/
	in cavity, 7 mode					in cavity, 10 mode				
Energy (cm^{-1})	3162	3466	3623	3655	3724	3187	3509	3644	3653	3754
VCI coeff (cavity)	0.03	0.72	0.68	/	0.22	0.02	0.71	0.52	0.19	0.19
VCI coeff (HB str)	0.16	0.66	0.72	0.08	0.02	0.14	0.55	0.61	0.31	0.09
VCI coeff (free str)	0.04	0.15	0.16	/	0.96	0.03	0.10	0.20	0.08	0.07
VCI coeff (sym str)	0.04	/	0.05	0.98	/	0.04	0.05	0.38	0.78	/
VCI coeff (bend overtone[b])	0.76	0.13	0.10	/	0.04	0.87	0.11	0.11	/	/

[a] fundamental frequency of the cavity mode
[b] bend overtone of the donor water
[c] complicated combination band involving water bend and low-frequency modes
[d] complicated combination band involving low-frequency modes only

Supplementary Table 3: Cavity vibrational self-consistent field/configuration interaction (cav-VSCF/VCI) state energies and leading vibrational configuration interaction (VCI) coefficient(s) for calculations in Supplementary Figure 2 with and without the inclusion of HB stretch. "/" indicates the VCI coefficients smaller than 0.005. "HB str", "free str", and "sym str" indicate the hydrogen bonded OH stretch, free OH stretch, and symmetric OH stretch respectively.

	in cavity, with HB stretch					in cavity, without HB stretch			
Energy (cm^{-1})	1602	3162	3514	3579	3724	1613	3210	3546	3821
VCI coeff (cavity)	0.01	0.01	0.71	0.70	0.09	0.01	/	0.99	0.06
VCI coeff (HB str)	0.01	0.15	0.67	0.70	0.08	/	/	/	/
VCI coeff (free str)	0.01	0.03	0.11	0.04	0.98	0.01	0.03	0.05	0.99
VCI coeff (sym str)	/	0.04	/	0.06	/	/	0.03	0.01	0.10
VCI coeff (bend overtone[a])	/	0.76	0.11	0.10	0.05	/	0.99	/	0.03
VCI coeff (bend fund[b])	0.99	/	0.01	0.01	/	0.99	/	0.01	0.01

[a] bend overtone of the donor water
[b] bend fundamental of the donor water

Supplementary Table 4: Cavity vibrational self-consistent field/configuration interaction (cav-VSCF/VCI) state energies and leading vibrational configuration interaction (VCI) coefficient(s) under different values of the light-matter coupling factor g with the cavity mode polarized along the O-O axis. "/" indicates the VCI coefficients smaller than 0.005. "HB str", "free str", and "sym str" indicate the hydrogen bonded OH stretch, free OH stretch, and symmetric OH stretch respectively.

Cavity mode frequency $\omega = 3547$ cm^{-1}								
Coupling factor g	0.00				0.001			
Energy (cm^{-1})	3162	3547	3547	3716	3162	3531	3563	3717
VCI coeff (cavity)	/[c]	/	1.0	/	<0.01	0.71	0.70	0.04
VCI coeff (HB str)	0.15	0.97	/	0.09	0.15	0.68	0.69	0.09
VCI coeff (free str)	0.04	0.16	/	0.99	0.03	0.09	0.05	0.99
VCI coeff (sym str)	0.04	/	0.07	/	0.04	/	0.05	/
VCI coeff (bend overtone[b])	0.76	0.15	/	0.05	0.76	0.11	0.11	0.05
Coupling factor g	0.002				0.005			
Energy (cm^{-1})	3162	3514	3579	3717	3162	3466	3623	3724
VCI coeff (cavity)	0.01	0.71	0.70	0.09	0.03	0.72	0.68	0.22
VCI coeff (HB str)	0.15	0.67	0.70	0.08	0.16	0.66	0.72	0.02
VCI coeff (free str)	0.03	0.11	0.04	0.98	0.04	0.15	0.16	0.96
VCI coeff (sym str)	0.04	/	0.06	/	0.04	/	0.05	/
VCI coeff (bend overtone[b])	0.76	0.11	0.10	0.05	0.76	0.13	0.10	0.04
Coupling factor g	0.008				0.01			
Energy (cm^{-1})	3162	3418	3658	3743	3161	3386	3671	3766
VCI coeff (cavity)	0.05	0.72	0.54	0.42	0.07	0.72	0.44	0.52
VCI coeff (HB str)	0.17	0.64	0.69	0.21	0.18	0.63	0.65	0.35
VCI coeff (free str)	0.03	0.18	0.43	0.87	0.03	0.20	0.59	0.77
VCI coeff (sym str)	0.04	/	0.16	/	0.05	/	0.18	/
VCI coeff (bend overtone[b])	0.77	0.15	0.11	0.01	0.78	0.17	0.11	<0.01

[a] fundamental frequency of the cavity mode

[b] bend overtone of the donor water

Supplementary Table 5: Cavity vibrational self-consistent field/configuration interaction (cav-VSCF/VCI) state energies and leading vibrational configuration interaction (VCI) coefficient(s) under different cavity mode frequencies with the cavity mode polarized along the O-O axis. "/" indicates the VCI coefficients smaller than 0.005. "HB str", "free str", and "sym str" indicate the hydrogen bonded OH stretch, free OH stretch, and symmetric OH stretch respectively.

Coupling factor g=0.005								
Cavity mode frequency	3250				3350			
Energy (cm^{-1})	3161	3230	3570	3719	3162	3320	3577	3720
VCI coeff (cavity)	0.16	0.95	0.24	0.08	0.07	0.94	0.32	0.10
VCI coeff (HB str)	0.18	0.20	0.95	/	0.17	0.30	0.93	0.08
VCI coeff (free str)	0.03	0.09	/	0.99	0.03	0.11	/	0.98
VCI coeff (sym str)	0.05	/	0.07	/	0.04	/	0.07	/
VCI coeff (bend overtone[a])	0.77	0.15	0.14	0.05	0.77	0.12	0.14	/
Cavity mode frequency	3450				3547			
Energy (cm^{-1})	3162	3403	3593	3721	3162	3466	3623	3724
VCI coeff (cavity)	0.04	0.87	0.46	0.14	0.03	0.72	0.68	0.22
VCI coeff (HB str)	0.16	0.45	0.85	/	0.16	0.66	0.72	0.02
VCI coeff (free str)	0.03	0.13	/	0.98	0.04	0.15	0.16	0.96
VCI coeff (sym str)	0.04	/	0.07	/	0.04	/	0.05	/
VCI coeff (bend overtone[a])	0.77	0.11	0.13	/	0.76	0.13	0.10	0.04
Cavity mode frequency	3650				3750			
Energy (cm^{-1})	3162	3504	3675	3737	3162	3521	3705	3791
VCI coeff (cavity)	0.02	0.49	0.70	0.51	0.02	0.34	0.34	0.87
VCI coeff (HB str)	0.16	0.83	0.49	0.14	0.16	0.91	0.25	0.25
VCI coeff (free str)	0.04	0.15	0.51	0.84	0.04	0.14	0.89	0.40
VCI coeff (sym str)	0.04	/	/	/	0.04	/	/	/
VCI coeff (bend overtone[b])	0.77	0.14	0.08	/	0.76	0.15	0.07	/

[a] bend overtone of the donor water

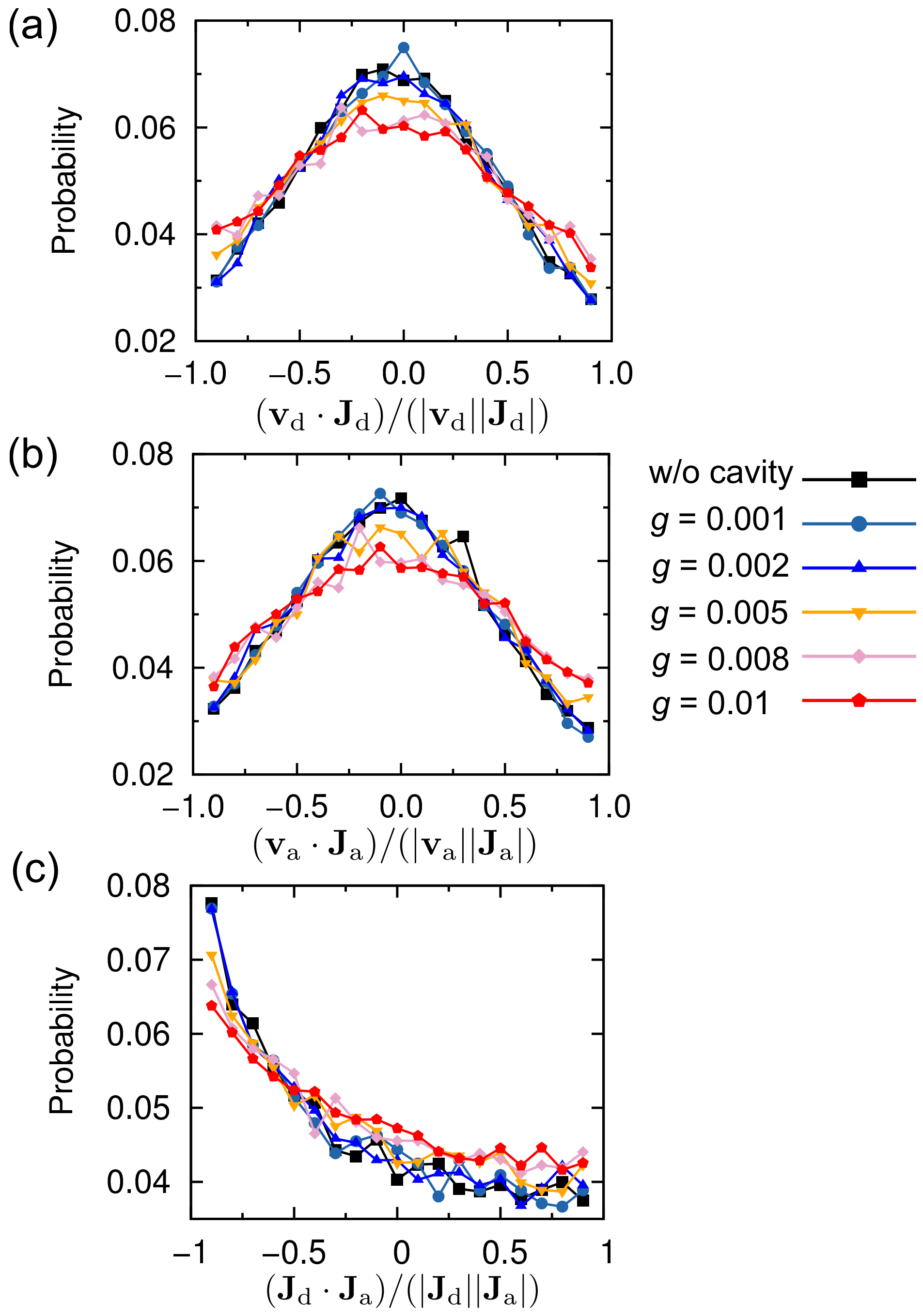

Supplementary Figure 3: Vibrational strong coupling effects on the velocity-angular momentum and angular momentum-angular momentum correlations. (a-b) Correlation between the relative center of mass velocity vector ($\mathbf{v}$) and the total angular momentum vector ($\mathbf{J}$) of either the donor ($\mathbf{v}_\mathrm{d}$,$\mathbf{J}_\mathrm{d}$) or the acceptor ($\mathbf{v}_\mathrm{a}$,$\mathbf{J}_\mathrm{a}$) fragment with different values of the light-matter coupling factor g for different cavity systems. (c) Correlation between the $\mathbf{J}_\mathrm{d}$ and $\mathbf{J}_\mathrm{a}$ vectors with different values of the light-matter coupling factor g for different cavity systems.

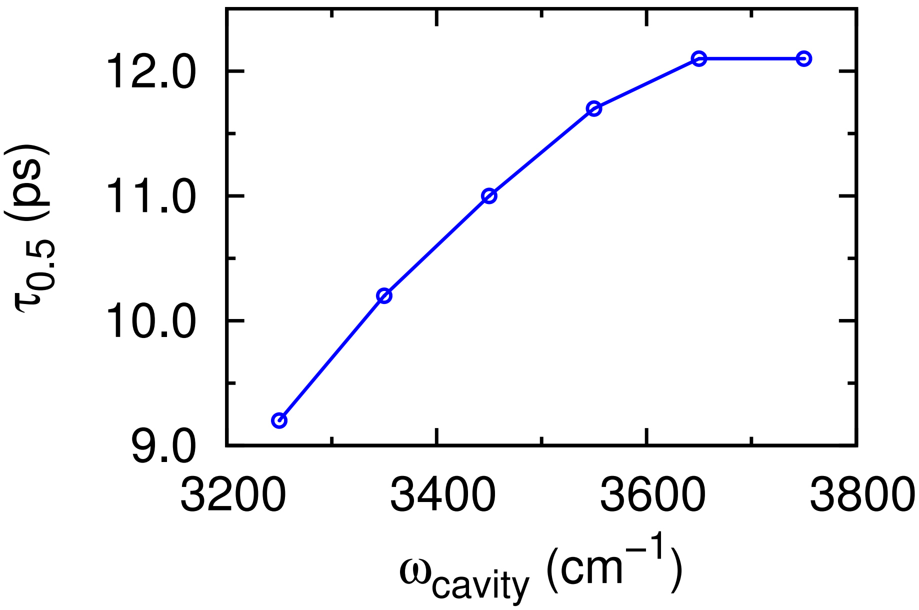

Supplementary Figure 4: Half lifetime ($\tau_{0.5}$) of $(H_2O)_2(\nu_{OH} = 1)$ for $(H_2O)_2$-cavity systems with different cavity frequencies (ω_{cavity}). ($\nu_{OH} = 1$) indicates one quanta excitation of the hydrogen-bonded OH stretch. The light-matter coupling factor g is 0.005.

Supplementary Table 6: Half lifetime of $(H_2O)_2(\nu_{OH} = 1)$ and dissociation rate at 25 ps among 20,000 trajectories for $(H_2O)_2$-cavity systems with different coupling strengths. The cavity frequency is set as 3547 cm^{-1}.

Light-matter coupling factor g	Half lifetime (ps)	Dissociation rate
0.0	13.5	82.5%
0.001	13.9	79.8%
0.002	13.6	80.9%
0.005	11.7	87.1%
0.008	9.6	92.8%
0.01	8.2	95.2%
0.015	5.6	98.9%

Supplementary Table 7: Half lifetime of $(H_2O)_2(\nu_{OH} = 1)$ and dissociation rate at 25 ps among 20,000 trajectories for $(H_2O)_2$-cavity systems with different cavity frequencies. The coupling strength factor g is set as 0.005.

Cavity frequency	Half lifetime (ps)	Dissociation rate
3250	9.2	95%
3350	10.2	92.4%
3450	11.0	89.5%
3547	11.7	87.1%
3650	12.1	85.9%
3750	12.1	85.9%

Supplementary Table 8: Vibrational product distributions for the dissociation of the water dimer for $(H_2O)_2$-cavity systems with different cavity frequencies. The coupling strength factor g is set as 0.005.

$(H_2O)_2(\nu_{OH} = 1) \rightarrow H_2O(n_1n_2n_3) + H_2O(n'_1n'_2n'_3)$						
	Cavity frequency (cm^{-1})					
$H_2O(n_1n_2n_3) + H_2O(n'_1n'_2n'_3)$	3250	3350	3450	3547	3650	3750
(000)+(000)	0.45	0.48	0.48	0.48	0.43	0.39
(000)+(010)	0.32	0.35	0.37	0.34	0.37	0.34
(000)+(100)	0.03	0.04	0.03	0.05	0.05	0.09
(000)+(001)	0.03	0.02	0.02	0.02	0.05	0.05
(000)+(020)	0.11	0.07	0.06	0.06	0.07	0.06
(010)+(010)	0.04	0.02	0.02	0.04	0.02	0.05
sum	0.98	0.98	0.99	0.99	0.99	0.94

(000) indicates the vibrational ground state, (010) and (020) indicates the bending fundamental and overtone states, (100) indicates the first-excited symmetric stretch state, and (001) indicates the first-excited asymmetric stretch state.

Supplementary References

(1) Carter, S.; Culik, S. J.; Bowman, J. M. Vibrational Self-consistent Field method for Many-mode Systems: A New Approach and Application to the Vibrations of CO Adsorbed on Cu(100). *J. Chem. Phys.* **1997**, *107*, 10458–10469.

(2) Carter, S.; Bowman, J. M.; Handy, N. C. Extensions and Tests of "Multimode": A Code to Obtain Accurate Vibration/Rotation Energies of Many-Mode Molecules. *Theor. Chem. Acc.* **1998**, *100*, 191–198.
